# Combined application of salicylic acid and silicon enhances photosynthetic capacity and yield in different dry-cultivated rice varieties: associations with osmotic adjustment substances and antioxidant enzyme activity

**DOI:** 10.3389/fpls.2026.1951660

**Published:** 2026-10-19

**Authors:** Zichen Liu, Pengfei An, Junjie Huang, Anran Sun, Qianqian Liu, Meikang Wu, Xiaoshuang Wei, Meiying Yang, Guan Wang, Zhihai Wu

**Affiliations:** 1 Faculty of Agronomy Jilin Agricultural University/Jilin Province Green and High Quality Japonica Rice Engineering Research Center, Changchun, China; 2 College of Life Science, Jilin Agricultural University, Changchun, China

**Keywords:** antioxidant enzyme, combined application of salicylic acid and silicon, dry-cultivated rice, osmotic adjustment substance, photosynthetic capacity

## Abstract

Single applications of silicon (Si) and salicylic acid (SA) can alleviate plant stress. However, the effects of their combined application on dry-cultivated rice and the differences between varieties remain unclear. A preliminary laboratory experiment established the optimal silicon fertilizer application rate at 45 kg ha^-1^. Subsequently, a preliminary experiment conducted in 2022 determined the optimal SA concentration at 1.0 mM. Based on these findings, four treatments were set up in 2023: control (CK), single application of Si (T1), single application of SA (T2), and Si + SA (T3). The results indicated that the combined application of Si and SA significantly enhanced both the grain yield and photosynthetic capacity of dry-cultivated rice. Under the T3 treatment, grain yield increased by 80.7% and 77.3% in cultivars SJ18 and HK88, respectively, compared with the CK. This yield improvement was primarily attributable to increases in two key yield components: the number of effective panicles (up by 17.6% and 29.3%, respectively) and the number of grains per panicle (up by 26.9% and 22.6%, respectively). The responses of the agronomic traits to the treatment were consistent with the yield trends. Physiologically, the combined application of Si + SA enhanced the photosynthetic capacity of the photosystem II (PSIl) system by improving chlorophyll fluorescence parameters, increasing antioxidant enzyme activities, and enhancing soluble sugar, soluble protein content, endogenous SA, and Si contents. The responses of different varieties to the combined application of Si + SA differed significantly. 10 days after spraying, the soluble sugar and protein content of HK88 increased by 36.1% and 22.7%, respectively, compared with CK, which were numerically greater than the corresponding increases observed in SJ18 (10.7% and 17.4%, respectively). This finding suggests that drought-resistant varieties may require greater accumulation of osmotic adjustment substances to alleviate drought stress. Therefore, the combined application of SA and Si is closely associated with increased levels of osmotic adjustment substances and endogenous SA and Si, as well as enhanced antioxidant enzyme activities. These coordinated physiological responses contribute to enhanced photosynthetic capacity of the PSII system, ultimately improving drought tolerance, dry matter accumulation, and grain yield in dry-cultivated rice. These relationships are further supported by significant positive correlations observed in our structural equation modeling analysis. This approach provides a new and effective way of alleviating drought stress and increasing the yield of dry-cultivated rice.

## 1 Introduction

Rice (*Oryza sativa* L.) is one of the major food crops in China. Improving rice production is of great significance for ensuring food security and improving people’s living standards in China and around the world (Yang et al., 2018). However, environmental factors such as high temperature, drought, and salinity often prevent rice from reaching its yield potential (Wei et al., 2024). In recent years, the frequency and severity of drought have increased worldwide, making drought a major constraint on global food production. Drought adversely affects the production and accumulation of dry matter in rice (Kumar et al., 2006). With increasing population and growing demand for water resources, the development of drought-resistant crops is crucial to prevent crop yield losses caused by drought (Kim et al., 2020). As a highly efficient, resource-saving, mechanized, climate-smart, and economically viable cultivation strategy (Chen et al., 2021), dry-cultivated rice has the potential to solve the shortage of labor-water resources and ensure the sustainable cultivation of rice. As a result, dry-cultivated rice is being considered an alternative to paddy rice.

The reproductive stage is considered the most drought-susceptible stage in plants. In cereal crops, particularly rice, this stage is highly sensitive to water limitation. Drought-induced reductions in carbon assimilation lead to increased production of reactive oxygen species (ROS) in leaf cells (Hussain et al., 2018). Excessive ROS accumulation promotes leaf senescence through the decomposition of photosynthetic pigments. Photosynthesis is generally positively correlated with crop yield. Impaired photosynthesis can adversely affect the growth and development of rice, thereby reducing its productivity (Urmi et al., 2023). Studies have found that salicylic acid (SA) plays an important role in regulating defense signal transduction and cell death (Yang et al., 2023).

SA is an endogenous small-molecule phenolic compound that can function as a signal sensor to regulate plant responses. It protects plant cells from the toxicity associated with ion accumulation and cell death by regulating processes such as antioxidant defense, nitrogen metabolism, photosynthesis, and water stress (Peng et al., 2021). Pretreatment of rice roots with SA can promote root growth, reduce ROS levels and membrane damage, increase the activities of superoxide dismutase (SOD), peroxidase (POD), and catalase (CAT), and increase glutathione content. It can also increase chlorophyll content and reduce malondialdehyde content. Exogenous application of SA alleviates oxidative stress in drought-stressed plants by regulating key enzymatic and non-enzymatic pathways (Prakash et al., 2021). In addition, SA is involved in glycolysis/TCA cycle, starch and sucrose metabolism, antioxidant defense, and other metabolic pathways, thereby mitigating the reductions in rice yield and grain weight caused by soil drought (Kimbembe et al., 2020). During drought stress, SA can improve the photosynthetic capacity of plants. Exogenous spraying of SA significantly increased the levels of photosystem I (PSI) and electron transfer rate (ETR) in *Toona ciliata*, which in turn promoted the recovery of photosystem II (PSIl) reaction-center activity and ultimately led to the increase of photosynthesis (Gao et al., 2023a).

Silicon (Si) is the second most abundant element in soil. However, it is not considered as essential for plant growth as other well-established inorganic nutrients, such as nitrogen, potassium, phosphorus, calcium, and magnesium (Pan et al., 2021). Nevertheless, Si is believed to play a beneficial role in plants exposed to various stresses (Krucky et al., 2025). Si application is an effective strategy for alleviating the adverse effects of drought stress (Chtouki et al., 2025). Si enhances plant resistance to various abiotic stresses by regulating the synthesis and accumulation of endogenous hormones. In abiotic stress-induced adversity, Si is involved in regulating various plant metabolic processes, including the generation of osmotic pressure and the regulation of antioxidant defense systems against ROS (Khan et al., 2021). Si can also promote plant growth by enhancing photosynthetic rates and nutrient absorption, improving plant defense response, and increasing tolerance to a range of stresses (Chaiwong et al., 2020). In rice, Si promotes the formation of young panicles and increases the grain-filling rate, thereby enhancing grain yield (Dhakate et al., 2022). In wheat (*Triticum aestivum* L.), Si accumulation alleviates drought stress by improving photosynthesis, transpiration, stomatal conductance, and water-use efficiency (Johnson et al., 2022).

Despite extensive research on the individual roles of SA and Si in enhancing crop drought tolerance, most studies have examined these factors independently, while the synergistic effects of their simultaneous application on the drought tolerance and yield of dry-cultivated rice remain largely unexplored. Notably, no studies have investigated the synergistic effects of aboveground SA spraying combined with belowground Si application on the drought tolerance and yield of dry-cultivated rice, necessitating further research. Furthermore, the physiological mechanisms underlying this synergistic interaction—particularly how the combined application coordinately regulates osmotic adjustment, antioxidant defense, and photosynthetic capacity—have not been systematically elucidated. In this study, the first systematic evaluation of combined SA + Si application specifically in dry-cultivated rice under field-like pot conditions will be conducted. Chlorophyll fluorescence, antioxidant enzyme activity, osmotic adjustment substances, and endogenous SA and Si contents will be integrated into a unified mechanistic framework. Comparative analyses will also be conducted using rice varieties with contrasting levels of drought tolerance to provide insights into genotype-dependent responses to combined SA + Si treatment. Therefore, this study will focus on the effects of the combined application of exogenous SA and Si on the morphological characteristics, yield, chlorophyll fluorescence, antioxidant enzyme activity, and Si and SA contents of dry-cultivated rice.

## 2 Materials and methods

### 2.1 Experiment site

The pot experiment was conducted from 2022 to 2023 at the Green High-Quality Japonica Rice Engineering Research Center (125°39 E, 44°46 N) of Jilin Agricultural University in Changchun, Jilin Province, China. The 0–20 cm soil layer had a clay loam texture, with a pH value of 6.7. The available silicon content was 113.46 mg kg^-1^, indicating a medium silicon supply capacity. The soil contained 9.60 g kg^-1^ of organic carbon, 33.89 mg kg^-1^ of alkali-hydrolyzable nitrogen, 137.09 mg kg^-1^ of available potassium, and 29.42 mg kg^-1^ of available phosphorus. Over the past three years, the region recorded a mean daily temperature of 21.2 °C and cumulative rainfall of 674.6 mm during the entire crop growth period.

### 2.2 Experiment design and cultivation conditions

A randomized block design was used in the experiment. Two high-quality dry-cultivated rice varieties from Jilin Province, China, were selected as experimental materials: Suijing 18 (SJ18), which has strong drought tolerance, and Hongke 88 (HK88), which has weak drought tolerance (Wei et al., 2022). In 2022, six salicylic acid (SA) concentrations were evaluated: 0, 0.01, 0.50, 1.00, 1.50, and 2.00 mM, namely C1, C2, C3, C4, C5, and C6, respectively. The SA solutions at different concentrations were sprayed on the leaves at the seedling stage of dry-cultivated rice (three-leaf and one-heart stage). The pot barrels were 14 cm in height and 15 cm in diameter, with three holes per pot and five grains sown in each hole. SA concentrations under different concentration treatments were measured at seven time points: 3, 6, 9, 12, 24, 36, and 72 h. The optimal SA concentration for future experiments under these experimental conditions was determined based on changes in SA concentration under different concentration treatments at different time points. In 2023, based on the optimum SA concentration determined in 2022, four treatments were used: control (traditional cultivation mode without exogenous application of silicon-Si or SA, CK), single application of Si (T1), single application of SA (T2), and combined application of Si and SA (T3). In the preliminary screening conducted in 2022, 1.0 mM SA was selected as the optimum concentration because it induced the highest physiological responses, including endogenous SA content and antioxidant enzyme activities. Therefore, 1.0 mM SA was used as the working concentration in the subsequent experiments. SA was sprayed at the panicle initiation stage. The pot buckets were 22 cm in height and 30 cm in diameter, with three holes per pot and eight grains sown per hole. Each treatment was replicated three times.

The experiment was conducted in a rainfall-resistant greenhouse in 2022 and 2023. No standing water layer was maintained throughout the entire growing season. Changes in soil water potential were monitored using a soil water potential meter (SYS-TSS1, Sayas Technology Co., Ltd., Liaoning, China). When the soil water potential at a depth of 10–15 cm fell below -35 kPa, water was added to increase the soil water potential to-10 kPa. The application rates of nitrogen, phosphorus, and potassium fertilizers were the same across all treatments. Nitrogen was applied at 160 kg ha^-1^ using urea as the fertilizer source (pure N 46%); phosphorus was applied at 75 kg ha^-1^ using calcium superphosphate as the fertilizer source (P_2_O_5_ 12%); and potassium was applied at 75 kg ha^-1^ using potassium chloride as the fertilizer source (K_2_O 60%). Nitrogen, phosphorus, and potassium fertilizers were all applied once as basal fertilizers. Biouli Russian mineral silicon fertilizer (effective Si content equal to or greater than 72%) was used as the silicon source. Si was applied at a rate of 45 kg ha^-1^ (Jiang et al., 2023) as a one-time basal fertilizer application. All other cultivation practices were carried out in accordance with standard high-yield cultivation requirements.

### 2.3 Sampling and measurements

#### 2.3.1 Yield and components

At the maturity stage, the number of effective panicles per pot was recorded, and five plants with a similar average number of panicles were selected. After drying, the total number of grains per panicle, number of filled grains, 1000-grain weight, and grain-filling rate were measured. The rice quality and water content were measured after drying, and rice yield was calculated based on a standard water content of 13.5%.

#### 2.3.2 Agronomic traits index

Dry matter accumulation: At the mature stage, five plants were randomly sampled from each treatment to determine dry matter accumulation. The samples were dried in an oven at 105 °C for 30 minutes and then at 80 °C until a constant weight was achieved. The dry matter weight was measured using a one-hundredth balance.

For root length, three replicates were selected for each treatment. Three pots of rice were carefully rinsed with tap water and then with purified water, after which the roots were gently straightened. The distance from the base of each plant to the root tip was measured using a ruler.

For plant height, the distance from the base of each rice plant to the top leaf was measured using a tape measure, and the average height was calculated.

#### 2.3.3 Chlorophyll fluorescence determination

At 24 hours after SA application at the panicle initiation stage, three plants with uniform growth were selected from each treatment. Their flag leaves were measured using a portable chlorophyll fluorometer (PAM-2500, Heinz Walz GmbH, Effeltrich, Germany) on the marked leaves in each pot. Fluorescence parameters were measured under dark treatment at 20:00–23:00 on the night before the measurement date and under natural light from 9:00–12:00 the next morning. The measured parameters included the maximum photochemical (quantum) efficiency of photosystem II (PSII) (Fv/Fm), photochemical quantum yield (ΦPSII), non-photochemical quenching coefficient (qN), and electron transport rate. (ETR).

#### 2.3.4 Determination of soluble sugar and soluble protein contents

At the booting stage, Si, SA, and SA + Si were sprayed, and leaves were sampled at 2 h, 24 h, and 10 days after treatment, respectively. Then, 0.1 g of dried plant samples (after crushing and sieving) was weighed into 2 mL EP tubes, and 80 μL of 80% ethanol was added. After sealing, the tubes were boiled in an 80 °C water bath for 30 minutes. The soluble sugar content was measured by sulfuric acid–anthrone colorimetry with the absorbance determined at 620 nm (Zhang et al., 2006).

At the booting stage, Si, SA, and SA + Si treatments were sprayed, and leaves were sampled at 2 h, 24 h, and 10 days after treatment, respectively. For each sample, 0.1 g of leaf tissue was weighed, frozen in liquid nitrogen, ground into a fine powder, and thoroughly homogenized in 1 mL of TBS. The homogenate was centrifuged at 14,000 rpm for 5 min at 4 °C, and the resulting supernatant was collected as the total soluble protein extract. Protein concentration was determined using the Bradford protein assay (Bio-Rad), with bovine serum albumin as the standard. The total soluble protein content of each sample was calculated based on the protein concentration and the volume of the protein extract. The average value was calculated from three seedlings for each experiment (Watanabe et al., 2022).

#### 2.3.5 Determination of antioxidant enzyme activity

Leaf samples (0.5 g) were accurately weighed and mixed with 5 mL of 50 mM HEPES buffer (pH 7.8) containing 20% (v/v) glycerol, 1 mM EDTA, 1 mM ASA, 1 mM GSH, 5 mM MgCl_2_, and 1 mM DTT. The mixture was ground and extracted in an ice bath, homogenized at 4 °C, and centrifuged at 10,000g for 20 minutes. The resulting supernatant was collected and used for the analysis of various enzyme activities.

For the determination of superoxide dismutase (SOD), the nitroblue tetrazolium (NBT) photochemical reduction method was used. The reaction mixture (3.0 mL) consisted of 1.5 mL of 0.05 M HEPES buffer (pH 7.8), 0.3 mL of 130 mM methionine (Met), 0.3 mL of 750 mM nitroblue tetrazolium (NBT), 0.3 mL of 100 μM disodium ethylenediaminetetraacetate (EDTA-Na_2_), 0.3 mL of 60 μM riboflavin, and an appropriate amount of enzyme extract. For the blank control, the enzyme extract was replaced with an equivalent volume of buffer. After illumination at 25 °C and 4000 lx for 15 minutes, the blank control changed from yellow to blue. The reaction was immediately stopped in the dark, and the absorbance was measured at 560 nm immediately (Beauchamp and Fridovich, 1971).

For the determination of catalase (CAT), the reaction mixture consisted of 1.9 mL of 50 mM HEPES–phosphate buffer (pH 7.0), 1 mL of 22 mM H_2_O_2_, and 0.1 mL of enzyme solution. The reaction was performed at 25 °C. The kinetic change of OD_240_ was measured, and the measurement interval was 10 s (Islam et al., 2023).

The mixture for determining peroxidase (POD) consists of 1 mL of sodium phosphate buffer (pH 7.8), 0.95 mL of 0.2% guaiacol, 1 mL of 0.3% H_2_O_2_, and 0.05 mL aliquot of enzyme extract. The absorbance was read at 470 nm for 90 seconds with an interval of 30 seconds. One unit of POD activity was defined as the amount of enzyme required to cause the decomposition of 1 mg substrate at 470 nm (Shuochen et al., 2023).

Ascorbic acid peroxidase (APX) activity assay was performed using a commercial kit (Beijing Solebold Technology Co., Ltd., Beijing, China).

Determination of glutathione reductase (GR) activity was performed using a commercial kit (Beijing Solebold Technology Co., Ltd., Beijing, China).

#### 2.3.6 Determination of Si content in dry-cultivated rice

Si content was determined using a modified molybdenum-blue colorimetric method. Plant samples (0.3 g) were accurately weighed into porcelain crucibles and incinerated in a muffle furnace at 550 °C for 3 hours. After ashing, the ash was quantitatively transferred into a 100 mL plastic bottle using 50 mL of 0.08 M H_2_SO_4_ solution. Subsequently, 2 mL of 40% hydrofluoric acid was added, and the mixture was shaken for 1 hour and allowed to stand overnight. On the next day, 1 mL of the suspension was pipetted into a 50 mL centrifuge tube and diluted to 50 mL with 0.32% H_3_BO_3_ solution. Then, 1.5 mL of the diluted sample solution was transferred to a 10 mL centrifuge tube, and 1.5 mL of chromogenic reagent (prepared by mixing equal volumes of 0.08 M H_2_SO_4_ and 2% ammonium molybdate solution) was added. After thorough shaking, the mixture was allowed to react for 5 minutes. Subsequently, 1.5 mL of 3.3% tartaric acid and 1.5 mL of 0.4% ascorbic acid were added sequentially, with thorough shaking after each addition. After a further 10 minutes, the absorbance was measured at 811 nm using a UV-Vis spectrophotometer (Beijing Puyi Company, Beijing, China). The Si content (%) in the sample was calculated using Equation 1.

(1) $$Si\;\left(\%\right)\;=\;\left(\rho \times V\right)/m\;\times \;100\%$$

Where ρ represents the mass concentration of SiO_2_ (mg L^-1^) derived from the standard working curve, V is the total volume of the sample solution (L), and m is the dry weight of the sample (g) (Neumann et al., 2011).

#### 2.3.7 Determination of SA content in dry-cultivated rice

Free salicylic acid (SA) was extracted and quantitated essentially as described by Malamy et al. (1992). Briefly, 1 g of frozen tissue was ground in 3 mL of 90% methanol and centrifuged at 6000 rpm for 15 minutes. The pellet was back-extracted with 3 mL of 100% methanol and centrifuged. The methanol extracts were combined, centrifuged at 1500 to 2000 rpm for 10 minutes, and dried under vacuum at 40 °C. The extracts were then resuspended in either 2.5 mL of 5% trichloroacetic acid (for temperature-shift experiments) or 2.5–5 mL of water at 80 °C (for SA conjugate experiments). When water was used as the solvent, an equal volume of 0.2 M sodium acetate buffer (pH 4.5) was added, and the pH was adjusted to 1.0 to 1.5 with HCl before extraction. Free SA was extracted with two volumes of cyclopentane/ethyl acetate/isopropanol 50:50:1. When performed, back-extractions used two volumes of the same solvent. The organic extracts were dried under nitrogen and analyzed by HPLC as described by Malamy et al. (1992).

### 2.4 Statistical analysis

One-way analysis of variance (ANOVA) was performed using the F-test (p < 0.05), and the group means were separated by Duncan’s multiple range test. Statistical analyses were conducted using IBM SPSS Statistics 26 software. Figures were generated using Origin 2023 software. Pearson correlation analysis and plotting of the correlation heatmap and network were performed using the “gpairs” package in R 4.4.3.

## 3 Results

### 3.1 Effects of different concentrations of salicylic acid on the growth of rice seedlings in dry-cultivated rice

Exogenous spraying of SA at different time intervals and concentrations significantly affected the endogenous SA concentration in dry-cultivated rice. In both varieties, the SA content in the leaves under all tested concentrations reached its highest level at 24 h after spraying and subsequently decreased (**Figure 1**). Therefore, the changes in SA content and various antioxidant enzyme activities in the leaves of dry-cultivated rice were determined at 24 h after spraying with different SA concentrations in order to determine the optimal spraying concentration.

**Figure 1:**
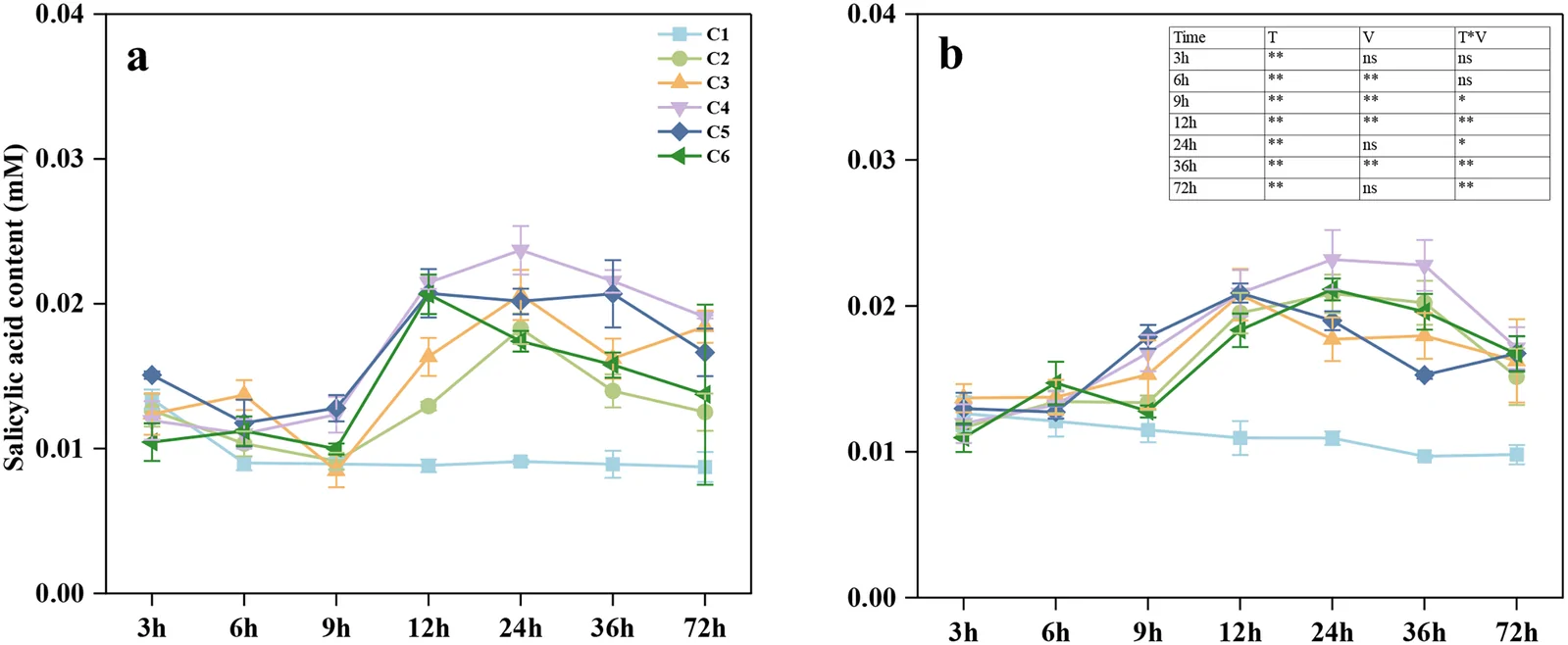
Salicylic acid (SA) content in the leaves of dry-cultivated SJ18 **(A)** and HK88 **(B)** rice under applications of SA at different concentrations and different time intervals. Data are means of replicates (n=3), and the vertical bars represent the standard error. C1, 0 mM SA concentration; C2, 0.01 mM SA concentration; C3, 0.5 mM SA concentration; C4, 1.0 mM SA concentration; C5, 1.5 mM SA concentration; C6, 2.0 mM SA concentration. T means treatment, V means variety, and T*V means the interaction between treatment and variety.

After spraying with different concentrations of exogenous SA, the endogenous SA concentration and antioxidant enzyme activities in the leaves of dry-cultivated rice were significantly affected (**Figure 2**). Compared with the 0 mM treatment, the endogenous SA content of the two varieties reached its highest level at the 1.0 mM concentration. Owing to the strong drought tolerance of SJ18, the SA content at the 0.5 and 1.5 mM concentrations was also close to that at the 1.0 mM treatment. All five antioxidant enzymes also reached their highest levels of enzymatic activity at an SA concentration of 1.0 mM (**Figure 2A**). Under the C4 treatment, the activities of superoxide dismutase (SOD) (**Figure 2B**), peroxidase (POD) (**Figure 2C**), catalase (CAT) (**Figure 2D**), glutathione reductase (GR) (**Figure 2E**), and ascorbic acid peroxidase (APX) (**Figure 2F**) were 12.9%, 46.7%, 23.7%, 370.9%, and 409.4% higher, respectively, than those under the C1 treatment. Therefore, spraying SA at a concentration of 1.0 mM in subsequent experiments can achieve the best experimental results.

**Figure 2:**
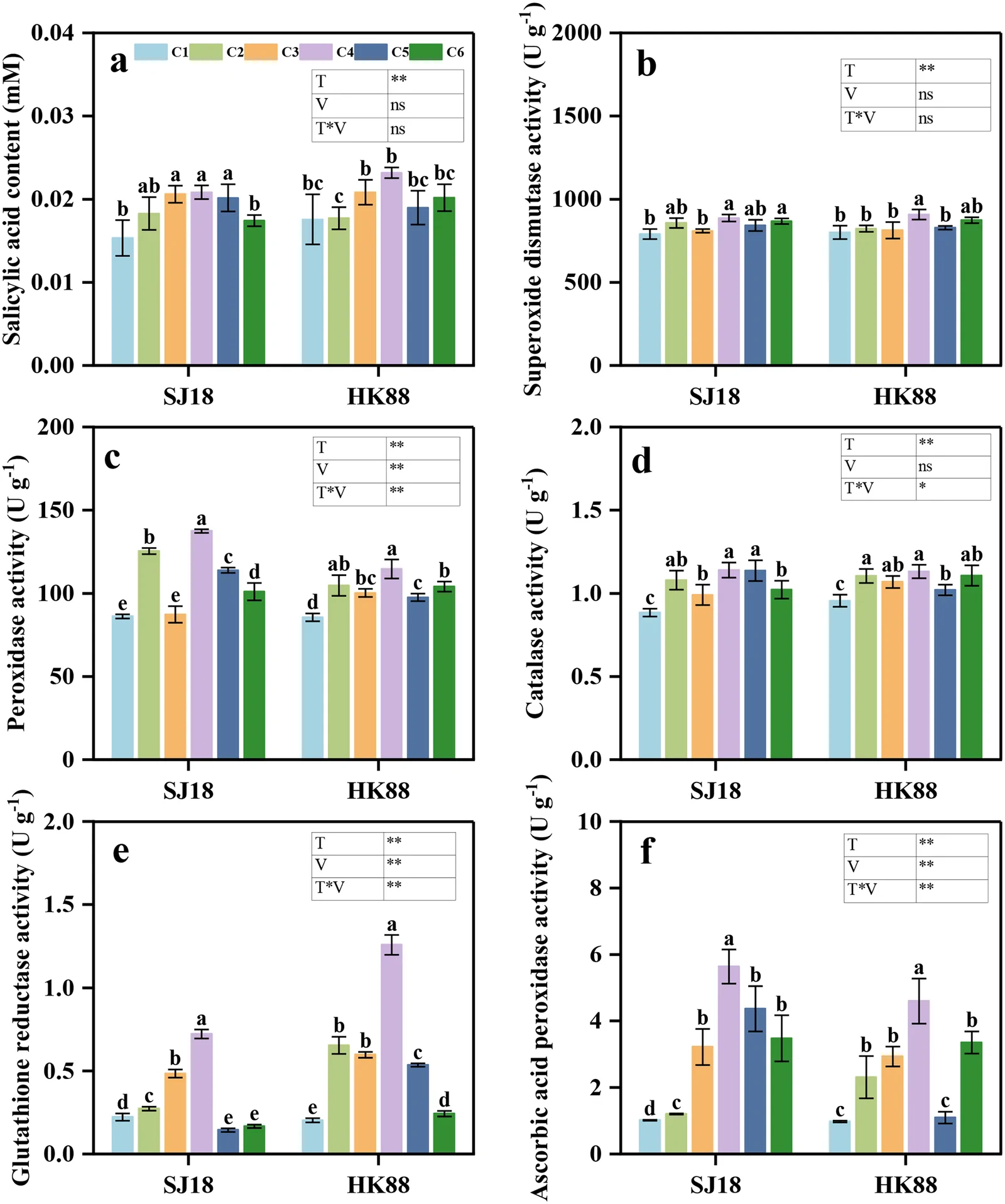
Effect of different concentrations of salicylic acid (SA) treatment for 24 h on SA content **(A)**, superoxide dismutase (SOD) **(B)**, peroxidase (POD) **(C)**, catalase (CAT) **(D)**, glutathione reductase (GR) **(E)** and ascorbic acid peroxidase (APX) **(F)** activities of dry-cultivated rice leaves. Data are means of replicates (n=3), and the vertical bars represent the standard error. Different letters above columns denote statistical significance at the P<0.05 level among treatments within each variety. C1, 0 mM SA concentration; C2, 0.01 mM SA concentration; C3, 0.5 mM SA concentration; C4, 1.0 mM SA concentration; C5, 1.5 mM SA concentration; C6, 2.0 mM SA concentration. T means treatment, V means variety, and T*V means the interaction between treatment and variety.

### 3.2 Yield and yield components

As shown in **Figure 3**, **Table 1**, different SA and silicon (Si) application methods had significant effects on the yield and yield components of dry-cultivated rice. Compared with the control (CK), both rice varieties showed varying degrees of yield increases under the T1 (Si), T2 (SA), and T3 (Si + SA) treatments, with T3 producing the greatest yield increase. Compared with the CK treatment, T3 increased the yields of SJ18 and HK88 by 80.7% and 76.2%, respectively, indicating that the combined application of different exogenous substances had a greater effect on the yield of SJ18.

**Figure 3:**
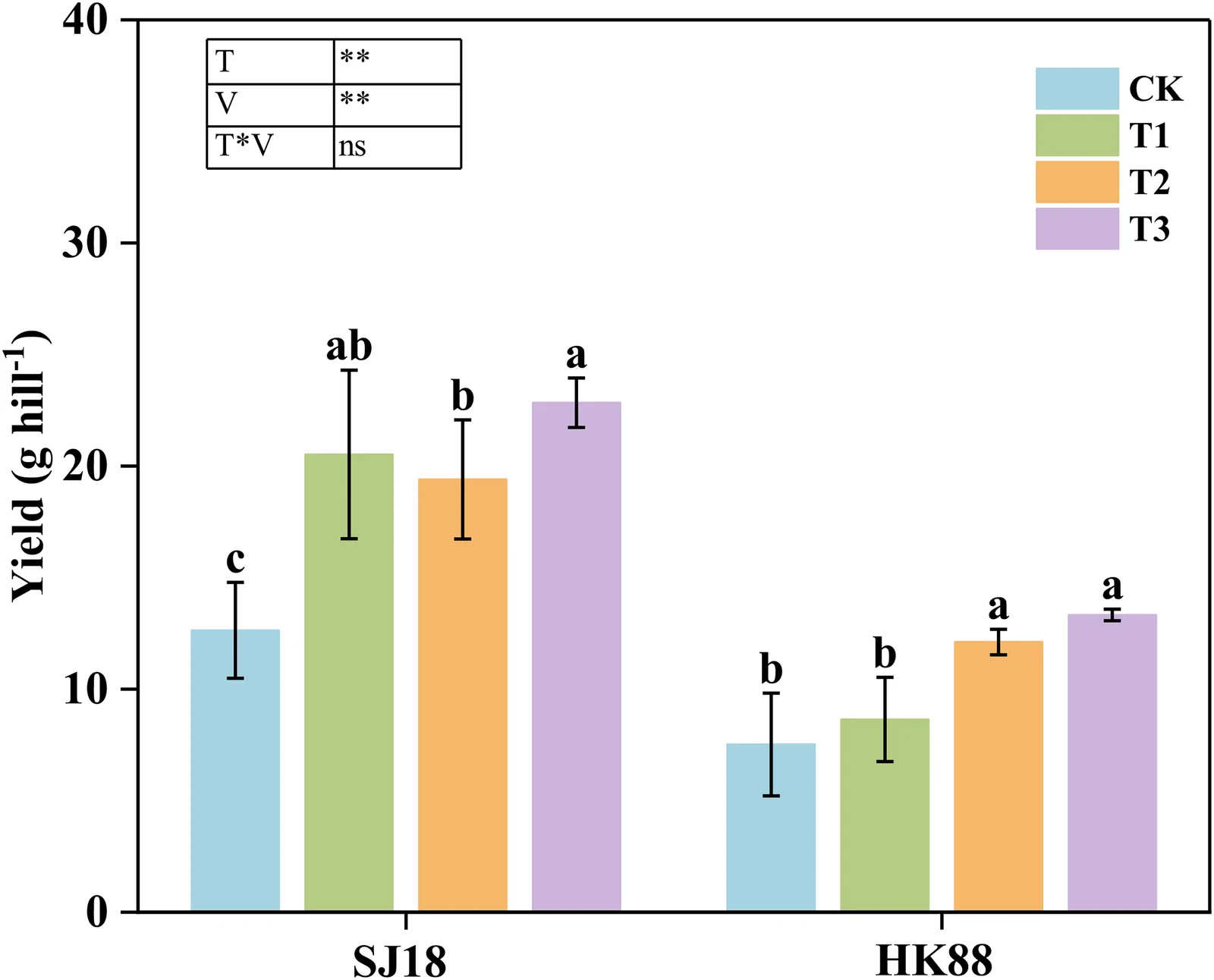
Effect of exogenous salicylic acid (SA) and silicon (Si) on yield in dry-cultivated rice in 2023. Data are means of replicates (n=3), and the vertical bars represent the standard error. Different letters above columns denote statistical significance at the P<0.05 level among treatments within each variety. Different letters above the columns indicate statistical significance at the p < 0.05 level for the same variety. CK, traditional cultivation mode without exogenous application of silicon and salicylic acid; T1, single application of silicon (45 kg ha^-1^); T2, single application of salicylic acid (1.0 mM); T3, silicon (45 kg ha^-1^) and salicylic acid (1.0 mM) combined application. T means treatment, V means variety, and T*V means the interaction between treatment and variety.

**Table 1:** Effect of exogenous salicylic acid (SA) and silicon (Si) on yield components in dry-cultivated rice.

Variety	Treatment	Panicles number	Spikelets panicle^−1^	Grain weight (g)	Grain filling (%)
SJ18	CK	13.33 ± 1.12b	60.67 ± 14.26b	21.59 ± 2.13a	0.78 ± 0.20b
	T1	13.00 ± 1.00b	75.44 ± 8.88a	22.72 ± 0.86a	0.92 ± 0.02a
	T2	15.00 ± 2.29a	66.78 ± 13.05ab	23.22 ± 1.64a	0.86 ± 0.10ab
	T3	15.67 ± 0.69a	77.00 ± 11.42a	22.04 ± 1.24a	0.85± 0.13ab
HK88	CK	9.67 ± 1.00b	50.78 ± 1.31b	20.01 ± 1.32b	0.84 ± 0.02a
	T1	10.00± 0.89b	61.67 ± 5.92a	17.55 ± 0.68c	0.80 ± 0.03b
	T2	14.33 ± 0.50a	62.50 ± 5.74a	20.40 ± 0.53b	0.69 ± 0.04c
	T3	12.50 ± 0.55ab	62.25 ± 5.38a	22.01 ± 0.99a	0.83 ± 0.05ab
	T	**	ns	ns	ns
	V	**	ns	**	ns
	T*V	ns	ns	ns	ns

Mean values followed by different letters in the same column are signiﬁcantly different from each other (p < 0.05). One-way analysis of variance (ANOVA) was performed by the F-test (p < 0.05), and the group means were separated by Duncan’s multiple range test. The data represent the means of three replicates (n = 3). CK, traditional cultivation mode without exogenous application of silicon and salicylic acid; T1, single application of silicon (45 kg ha^-1^); T2, single application of salicylic acid (1.0 mM); T3, silicon (45 kg ha^-1^) and salicylic acid (1.0 mM) combined application. T means treatment, V means variety, and T*V means the interaction between treatment and variety. * significant at P< 0.05; ** significant at P< 0.01; “ns” significant at “not significant”.

The differences in yield caused by different exogenous substances were mainly attributable to the number of grains per panicle and the number of effective panicles. The responses of 1000-grain weight and grain filling to the different treatments varied between the two varieties (**Table 1**). The main reason for the yield increase resulting from the combined application of SA and Si was the increase in the number of panicles per hole and the number of grains per panicle. Compared with the CK treatment, the T3 treatment increased the number of panicles per hole and the number of grains per panicle by 17.6% and 26.9%, respectively, in SJ18. The number of panicles per hole and the number of grains per panicle increased by 29.3% and 22.6%, respectively, under the T3 treatment of HK88. Although the numerical increase in the number of panicles per hole was greater in HK88 than in SJ18 under T3, the variety × treatment interaction was not significant for this trait (**Table 1**). Therefore, the yield improvement was mainly associated with increases in the number of panicles per hole and the number of grains per panicle in both varieties. The effects of exogenous substances on 1000-grain weight and grain filling varied among treatments. However, the variety × treatment interaction was not significant for these traits (**Table 1**). The 1000-grain weight of SJ18 showed no significant differences among different treatments.

### 3.3 Growth and biomass of dry-cultivated rice

As shown in **Figure 4**, the four different treatment combinations had significant effects on the dry weight, plant height, and root length of dry-cultivated rice. Compared with CK, the combined application of SA and Si significantly increased dry matter accumulation, plant height, and root length of dry-cultivated rice. The overall trend of underground dry weight, plant height, and root length under different treatment combinations was T3 > T1 > T2 > CK. Compared with CK, the shoot dry weight of SJ18 under T1, T2, and T3 treatments increased by 34.2%, 24.2%, and 40.0% (**Figure 4A**), respectively, while the root dry weight increased by 2.5%, 19.0%, and 48.9% (**Figure 4B**), respectively. The shoot dry weight of HK88 under T1, T2, and T3 treatments increased by 30.9%, 24.0%, and 39.1% (**Figure 4A**), respectively, while the root dry weight increased by 42.0%, 20.5%, and 53.4% (**Figure 4B**), respectively. Under the T3 treatment, the plant height and root length of SJ18 increased by 17.8% and 43.8% (**Figures 4C, D**), respectively, compared with CK. Meanwhile, the plant height and root length of HK88 increased by 20.2% and 57.7% (**Figures 4C, D**), respectively, compared with CK. Both varieties exhibited increased dry matter, plant height, and root length under T3 compared with CK. Although the numerical increases were generally greater in HK88, the variety × treatment interaction was not significant for these traits (**Figure 4**). Therefore, the growth-promoting effect of SA + Si was consistent across the two varieties.

**Figure 4:**
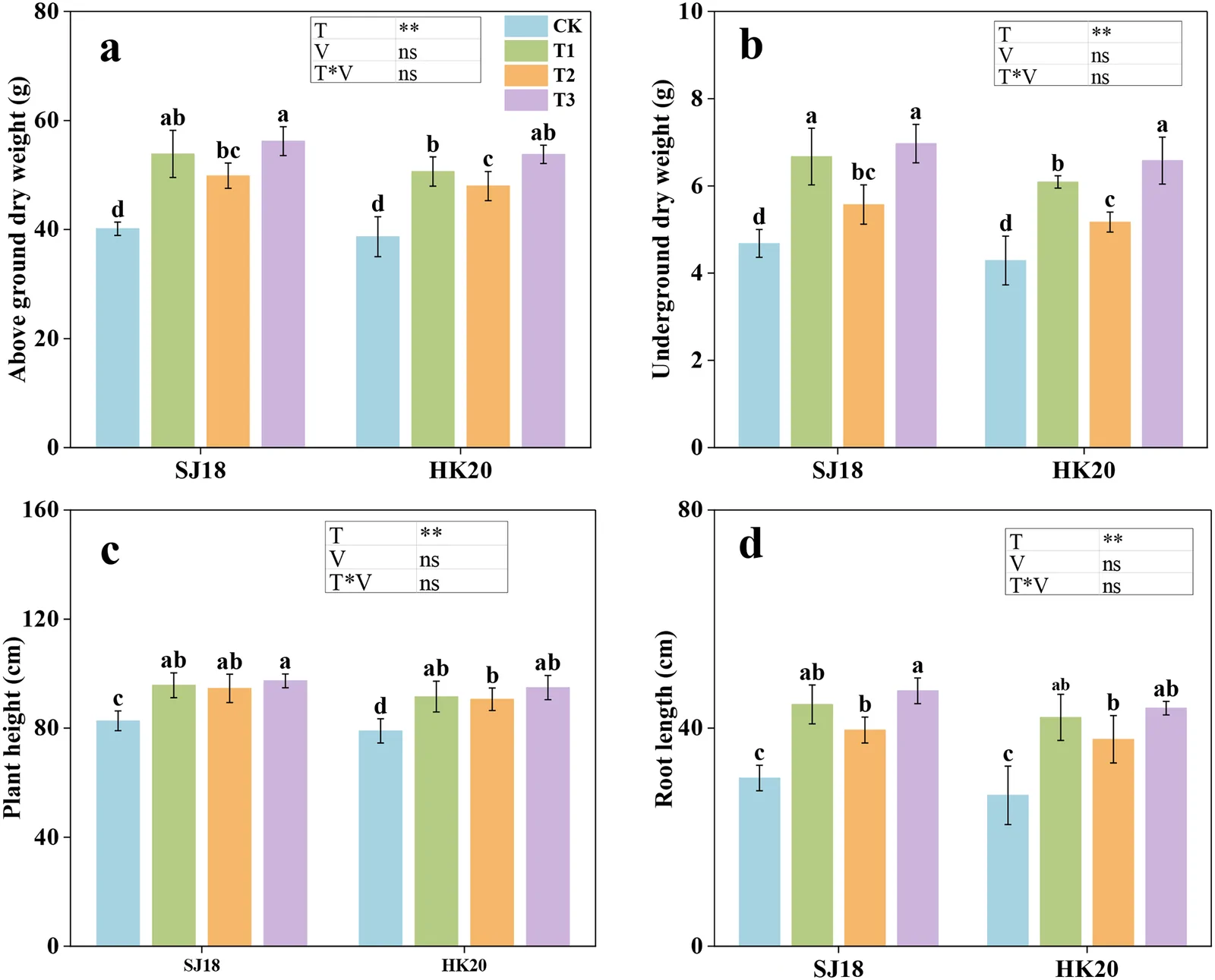
Effects of exogenous salicylic acid (SA) and silicon (Si) on above ground dry weight **(A)**, underground dry weight **(B)**, plant height **(C)**, and root length **(D)** of dry-cultivated rice. Data are means of replicates (n=3), and the vertical bars represent the standard error. Different letters above columns denote statistical significance at the P<0.05 level among treatments within each variety. Different letters above the columns indicate statistical significance at the p < 0.05 level for the same variety. CK, traditional cultivation mode without exogenous application of silicon and salicylic acid; T1, single application of silicon (45 kg ha^-1^); T2, single application of salicylic acid (1.0 mM); T3, silicon (45 kg ha^-1^) and salicylic acid (1.0 mM) combined application. T means treatment, V means variety, and T*V means the interaction between treatment and variety.

### 3.4 Chlorophyll fluorescence

Chlorophyll fluorescence is an accurate indicator of the photosynthetic capacity and efficiency of plant leaves, as well as the degree of environmental stress they experience. After exogenous spraying of SA, Si, and SA + Si, the Fv/Fm values of the two dry-cultivated varieties recovered to 0.7-0.85, significantly improving the maximum photochemical efficiency of PSII and enhancing the photosynthetic capacity of the two dry-cultivated varieties. The combined application of SA and Si had the best effect. Compared with the CK treatment, the Fv/Fm values of the T1, T2, and T3 treatments of SJ18 increased by 14.7%, 28.3%, and 31.0%, respectively, while those for HK88 increased by 11.7%, 12.1%, and 19.2%, respectively (**Table 2**). The actual photochemical quantum yield of the two varieties also increased significantly after the application of exogenous substances, particularly with the combination of SA and Si, which was consistent with the trend of Fv/Fm values. In contrast, the NPQ and ETR values of the two varieties decreased significantly after the application of exogenous substances, while the non-photochemical quenching coefficient NPQ value decreased. Under the T3 treatment, the NPQ values of SJ18 and HK88 decreased by 67.6% and 38.0%, respectively, compared with CK. These results indicate that exogenous substances alleviated drought-related photosynthetic inhibition in both varieties.

**Table 2:** Significance analysis of differences in chlorophyll fluorescence parameters among different treatments after 24 hours of exogenous salicylic acid (SA) spraying.

Variety	Treatment	Fv/Fm	ΦPSII	NPQ	ETR
SJ18	CK	0.65 ± 0.08d	0.41 ± 0.06e	1.82 ± 0.62a	31.27 ± 4.71ab
	T1	0.77 ± 0.03c	0.49 ± 0.01b	0.44 ± 0.06d	18.47 ± 0.30d
	T2	0.83 ± 0.02a	0.50 ± 0.02b	0.52 ± 0.06c	17.90 ± 0.58de
	T3	0.85 ± 0.01a	0.53 ± 0.01a	0.59 ± 0.19bc	17.50 ± 0.53e
HK88	CK	0.67 ± 0.02d	0.36 ± 0.02f	0.21 ± 0.04ef	34.45 ± 4.38a
	T1	0.75 ± 0.01c	0.43 ± 0.03de	0.17 ± 0.03f	29.24 ± 2.43b
	T2	0.75 ± 0.01c	0.43 ± 0.01d	0.16 ± 0.03f	28.41 ± 1.27b
	T3	0.80 ± 0.01b	0.47 ± 0.02bc	0.13 ± 0.02h	24.40 ± 1.25c
	T	**	**	**	**
	V	ns	**	**	**
	T*V	ns	ns	**	*

Mean values followed by different letters in the same column are signiﬁcantly different from each other (p < 0.05). One-way analysis of variance (ANOVA) was performed by the F-test (p < 0.05), and the group means were separated by Duncan’s multiple range test. The data represent the means of three replicates (n = 3). CK, traditional cultivation mode without exogenous application of silicon and salicylic acid; T1, single application of silicon (45 kg ha^-1^); T2, single application of salicylic acid (1.0 mM); T3, silicon (45 kg ha^-1^) and salicylic acid (1.0 mM) combined application. Fv/Fm, the maximum photochemical (quantum) efficiency of photosystem II (PSII); ΦPSII, the photochemical quantum yield; NPQ, the non-photochemical quenching coefficient; ETR, electron transport rate. T means treatment, V means variety, and T*V means the interaction between treatment and variety. * significant at P< 0.05; ** significant at P< 0.01; “ns” significant at “not significant”.

### 3.5 Soluble sugar and soluble protein content

The effects of different exogenous substances on soluble sugar and protein contents were significant, although the significant differences varied between varieties (**Figure 5**). After 10 days of treatment with different exogenous substances, the soluble sugar contents of SJ18 and HK88 under the T3 treatment increased by 10.7% and 36.1% (**Figures 5A, B**), respectively, compared with CK. Compared with the CK, the soluble protein content of SJ18 under the T3 treatment increased by 21.2%, 27.8%, and 17.4% after 2 hours, 24 hours, and 10 days of different exogenous substance treatments, respectively (**Figure 5C**). Similarly, the soluble protein content of HK88 under the T3 treatment increased by 11.0%, 15.5%, and 22.7% after 2 hours, 24 hours, and 10 days of different exogenous substance treatments, respectively (**Figure 5D**). Both varieties indicated increases in soluble sugar and soluble protein content after exogenous application, with the highest values generally observed under T3. Although HK88 exhibited numerically greater increases at 10 days, the variety × treatment interaction was not significant for these traits (**Figure 5**). Therefore, the osmotic adjustment response to SA + Si was generally consistent between the two varieties.

**Figure 5:**
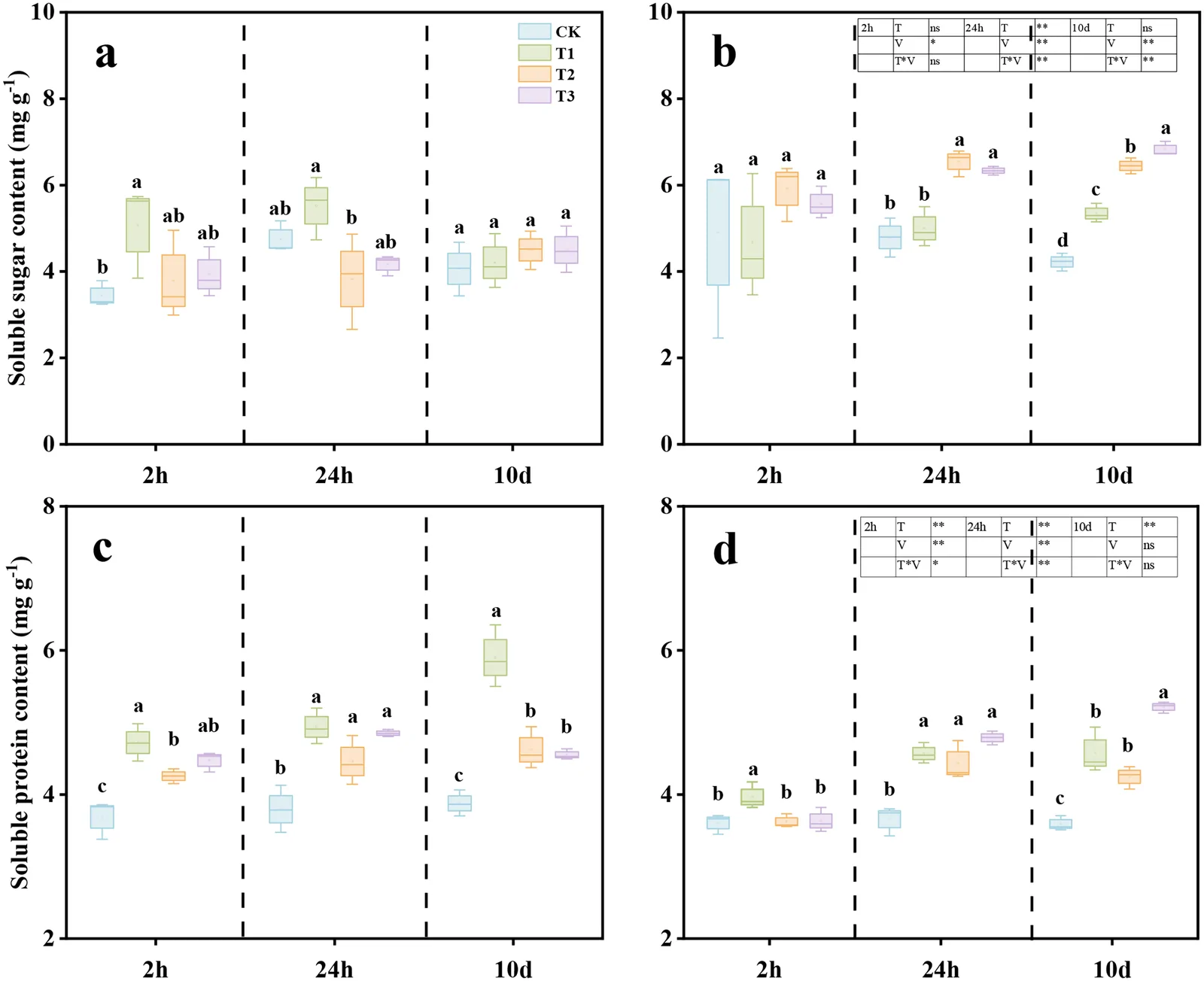
Effects of exogenous salicylic acid (SA) and silicon (Si) on soluble sugar and soluble protein content in dry-cultivated SJ18 [**(A, C)**, respectively] and HK88 [**(b, d)**, respectively)] rice. Data are means of replicates (n=3), and the vertical bars represent the standard error. Different letters above columns denote statistical significance at the P<0.05 level among treatments within each variety. CK, traditional cultivation mode without exogenous application of silicon and salicylic acid; T1, single application of silicon (45 kg ha^-1^); T2, single application of salicylic acid (1.0 mM); T3, silicon (45 kg ha^-1^) and salicylic acid (1.0 mM) combined application. T means treatment, V means variety, and T*V means the interaction between treatment and variety.

### 3.6 Antioxidant enzyme activity

As shown in **Figure 6**, the responses of superoxide dismutase (SOD), peroxidase (POD), catalase (CAT), glutathione reductase (GR), and ascorbic acid peroxidase (APX) activities in the two varieties to the different exogenous substance treatments were generally consistent. After the application of exogenous substances, the activities of all five antioxidant enzymes increased to varying degrees, while the T3 treatment exhibited the highest activities at 2 hours, 24 hours, and 10 days after different exogenous substance treatments. Overall, enzyme activities under the different exogenous substances increased with the application time, following the trend of 10 d > 24 h > 2 h (except for the SOD activity of SJ18 at 2 h after application and that of HK88 at 10 d after application). After 10 days of treatment with the different exogenous substances, the activities of SOD, POD, GR, CAT, and APX of SJ18 under the T3 treatment increased by 40.8%, 67.7%, 49.1%, 33.1%, and 41.5% (**Figure 6**), respectively, compared with the CK treatment. The activities of SOD, POD, GR, CAT, and APX of HK88 under the T3 treatment increased by 25.1%, 51.2%, 70.4%, 8.3%, and 37.3% (**Figure 6**), respectively, compared with the CK treatment. Differences were also observed in the activities of the five antioxidant enzymes between the two varieties. Both varieties exhibited increased antioxidant enzyme activities under exogenous treatments, with the highest activities observed under T3. Although the numerical increases were sometimes larger in HK88, the variety × treatment interaction was not significant for most enzymes (**Figure 6**). These results indicate the antioxidant response to SA + Si was largely consistent between the two varieties.

**Figure 6:**
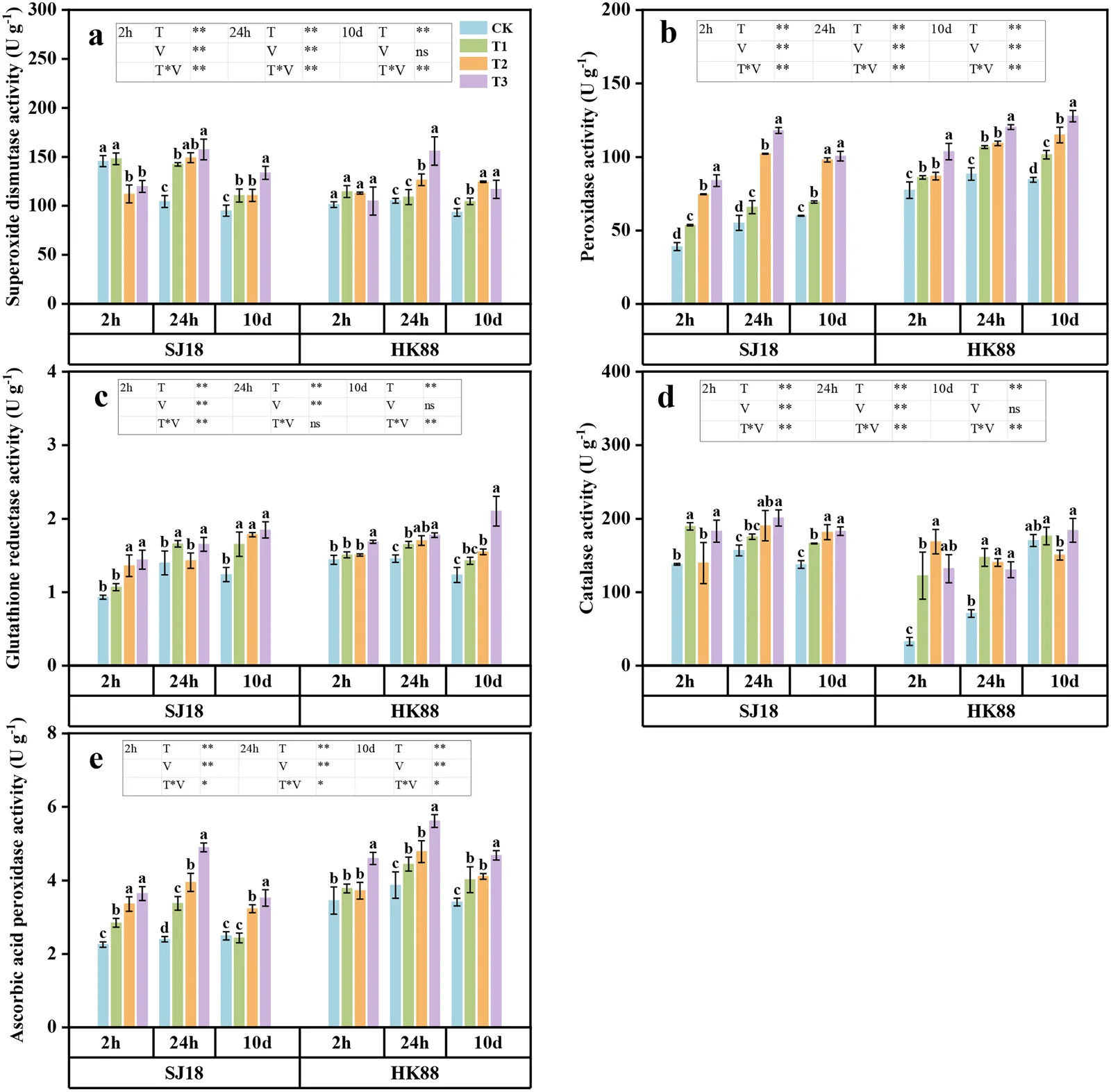
Effects of exogenous salicylic acid (SA) and silicon (Si) on superoxide dismutase (SOD) **(A)**, peroxidase (POD) **(B)**, glutathione reductase (GR) **(C)**, catalase (CAT) **(D)**, and ascorbic acid peroxidase (APX) **(E)** activities in dry-cultivated rice. Data are means of replicates (n=3), and the vertical bars represent the standard error. Different letters above columns denote statistical significance at the P<0.05 level among treatments within each variety. CK, traditional cultivation mode without exogenous application of silicon and salicylic acid; T1, single application of silicon (45 kg ha^-1^); T2, single application of salicylic acid (1.0 mM); T3, silicon (45 kg ha^-1^) and salicylic acid (1.0 mM) combined application. T means treatment, V means variety, and T*V means the interaction between treatment and variety.

### 3.7 Contents of endogenous salicylic acid and silicon

The endogenous Si and SA contents in the stems, leaves, and panicles of dry-cultivated rice were significantly affected by the different exogenous substances (**Table 3**; **Figure 7**). The Si content in the stems, leaves, and panicles followed the trend T3 > T1 > T2 > CK, with the combined application of SA + Si significantly increasing Si content in all three plant parts. Compared with CK, the Si content in the stems of SJ18 under T1, T2, and T3 increased by 159.0%, 66.1%, and 160.7%, respectively. The Si content in the leaves under T1, T2, and T3 increased by 496.4%, 182.7%, and 570.5%, respectively. The Si content in panicles under T1, T2, and T3 increased by 385.8%, 218.1%, and 542.8%, respectively. Compared with CK, the Si content in the stems of HK88 under T1, T2, and T3 increased by 77.7%, 70.0%, and 516.2%, respectively; the Si content in the leaves increased by 65.2%, 56.7%, and 351.7%, respectively; and the Si content in the panicles increased by 55.0%, 45.1%, and 371.2%, respectively. The SA content showed a trend consistent with that of Si content in each plant part. Under the T3 treatment, the SA content of the two varieties at 2 hours, 24 hours, and 10 days increased by 36.0%, 39.1%, and 28.5%, respectively, compared with CK. A significant variety × treatment interaction was observed for Si content in the stems, leaves, and panicles (**Table 3**), indicating that the responses to exogenous substances differed between the two varieties. HK88 exhibited greater increases in Si content, particularly under T3.

**Table 3:** Effects of different exogenous substances on endogenous silicon (Si) content in stems, leaves, and panicles of dry-cultivated rice.

Variety	Treatment	Si content in stems (mg g^-1^)	Si content in leaves (mg g^-1^)	Si content in ear (mg g^-1^)
SJ18	CK	128.12 ± 10.21c	41.34 ± 11.66d	66.48 ± 8.51d
	T1	331.79 ± 10.75a	246.56 ± 12.19b	322.97 ± 8.39b
	T2	212.73 ± 8.04b	116.86 ± 6.73c	211.44 ± 3.37c
	T3	334.04 ± 2.74a	277.19 ± 0.85c	427.36 ± 0.51a
HK88	CK	54.56 ± 2.16d	84.58 ± 0.88d	64.68 ± 2.47d
	T1	96.97 ± 2.15b	139.74 ± 0.69b	100.22 ± 4.12b
	T2	92.76 ± 1.65c	132.51 ± 1.76c	93.85 ± 3.31c
	T3	336.21 ± 9.63a	382.01 ± 11.18a	304.74 ± 11.89a
	T	**	**	**
	V	**	**	**
	T*V	**	**	**

Mean values followed by different letters in the same column are signiﬁcantly different from each other (p < 0.05). One-way analysis of variance (ANOVA) was performed by the F-test (p < 0.05), and the group means were separated by Duncan’s multiple range test. The data represent the means of three replicates (n = 3). CK, traditional cultivation mode without exogenous application of silicon and salicylic acid; T1, single application of silicon (45 kg ha^-1^); T2, single application of salicylic acid (1.0 mM); T3, silicon (45 kg ha^-1^) and salicylic acid (1.0 mM) combined application. T means treatment, V means variety, and T*V means the interaction between treatment and variety. * significant at P< 0.05; ** significant at P< 0.01.

**Figure 7:**
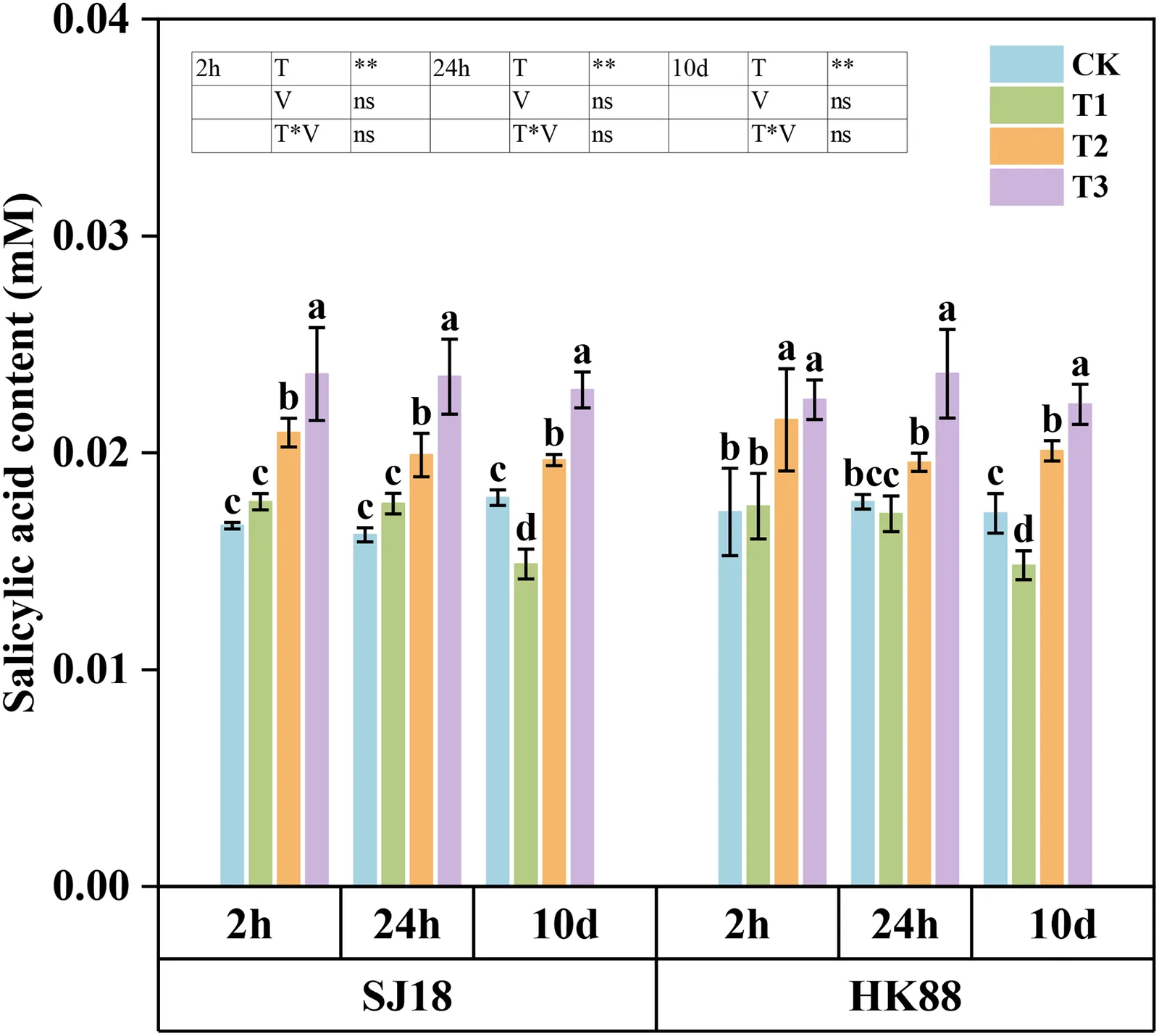
Effects of exogenous salicylic acid (SA) and silicon (Si) on endogenous salicylic acid (SA) content in leaves of dry-cultivated rice. Data are means of replicates (n=3), and the vertical bars represent the standard error. Different letters above columns denote statistical significance at the P<0.05 level among treatments within each variety. CK, traditional cultivation mode without exogenous application of silicon and salicylic acid; T1, single application of silicon (45 kg ha^-1^); T2, single application of salicylic acid (1.0 mM); T3, silicon (45 kg ha^-1^) and salicylic acid (1.0 mM) combined application. T means treatment, V means variety, and T*V means the interaction between treatment and variety.

### 3.8 Analysis of yield-dry matter accumulation-chlorophyll fluorescence cascade effect

Correlation analysis indicated significant positive correlations between yield and yield components (except seed-setting rate), plant height, primary light energy conversion efficiency, actual photochemical quantum yield, and endogenous Si content in various plant parts (**Figure 8**). Aboveground and underground dry matter weights were mainly associated with plant height, root length, primary light energy conversion efficiency, actual photochemical quantum yield, soluble protein content, and endogenous Si content in various plant parts. Antioxidant enzyme activity was positively correlated with chlorophyll fluorescence parameters. The structural equation model further elucidated the cascade effect between rice yield, dry matter accumulation, and chlorophyll fluorescence (**Figure 9**). Endogenous SA and Si contents were significantly positively associated with antioxidant enzyme activity (r = 0.835), while soluble sugar and protein contents were also positively correlated (r = 0.244). Further analysis indicated that the increase in antioxidant enzyme activity improved the chlorophyll fluorescence parameters (r = 0.805). The chlorophyll fluorescence parameters were positively correlated with yield, with a correlation coefficient of 0.754. These correlation and SEM results indicate statistically significant associations rather than causal effects. On the other hand, antioxidant enzyme activity was positively associated with chlorophyll fluorescence parameters, which were, in turn, positively correlated with yield, indicating an indirect rather than direct association with yield. Soluble sugar and protein contents were also associated with dry matter accumulation.

**Figure 8:**
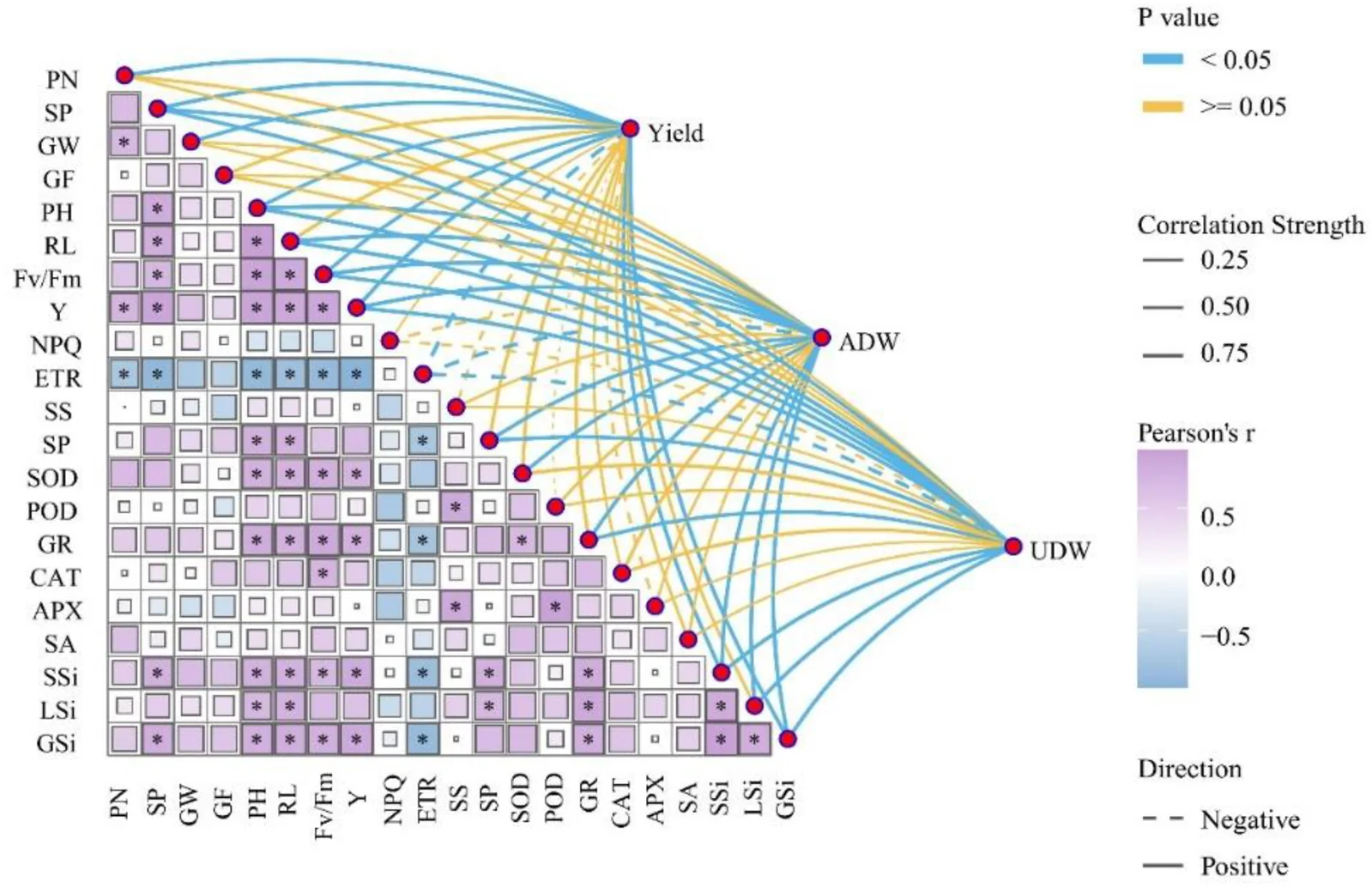
Correlation analysis of yield, aboveground biomass, and belowground biomass with various indicators. PN, panicles number; SP, spikelets panicle^−1^; GW, grain weight; GF, grain filling; PH, plant height; RL, root length; ADW, above ground dry weight; UDW, underground dry weight; Fv/Fm, the minimum photochemical efficiency; ΦPSII, the photochemical quantum yield; NPQ, the non-photochemical quenching coefficient; ETR, electron transport rate; SS, soluble sugar; SP, soluble protein; SOD, superoxide dismutase; POD, peroxidase; CAT, catalase; APX, ascorbate peroxidase; GR, glutathione reductase; SA, salicylic acid; SSi, silicon content in stems; LSi, silicon content in leaves; GSi, silicon content in ear. * significant at P< 0.05; ** significant at P< 0.01. The size of the squares represents the strength of the correlation.

**Figure 9:**
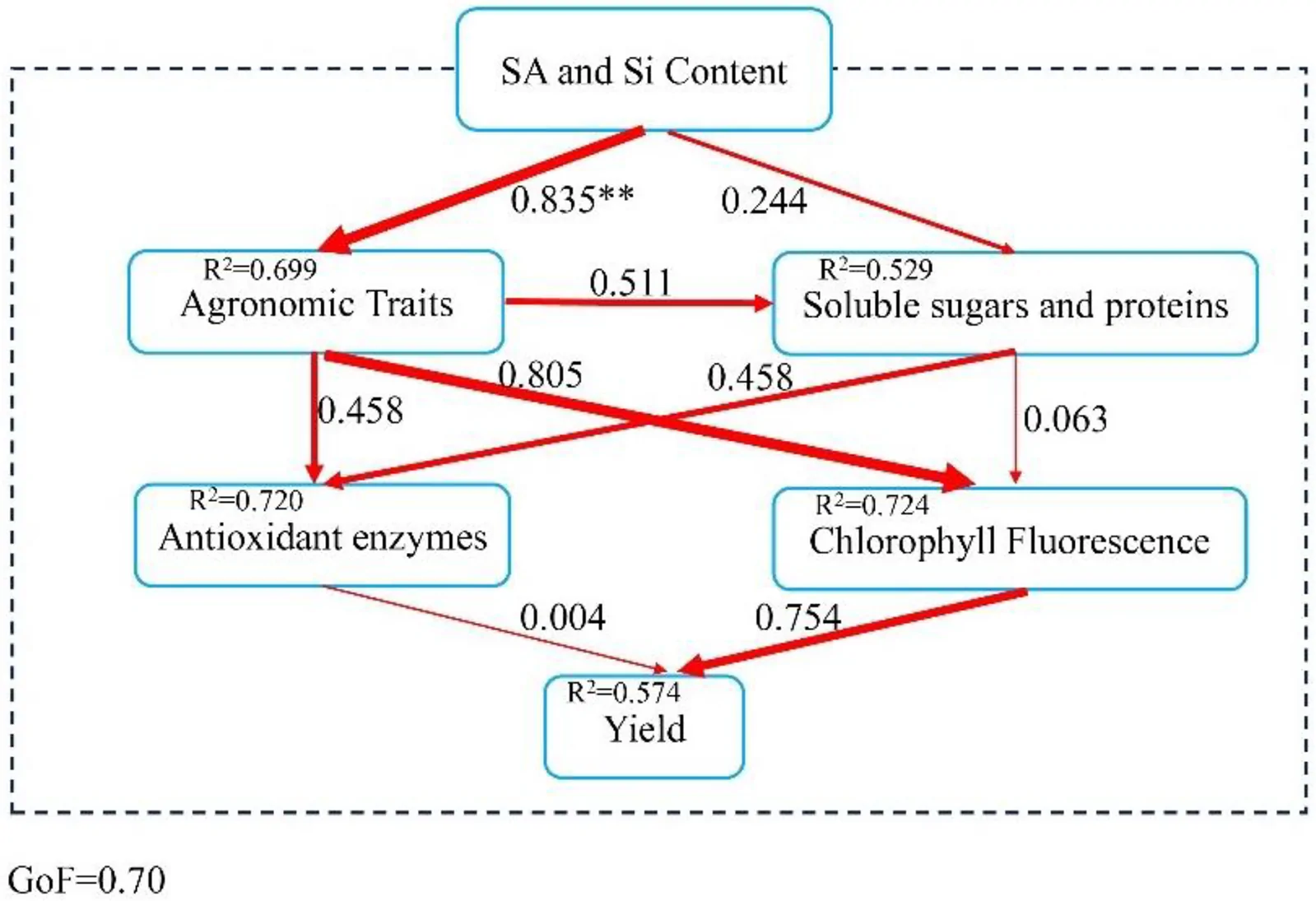
Based on experimental data, a structural equation model was used to investigate the direct and indirect effects of increased endogenous SA and Si content on yield through antioxidant enzyme activity and soluble sugar and protein content. The direct path from antioxidant enzyme activity to yield is negligible (β = 0.004); antioxidant enzyme activity is mainly associated with yield indirectly through chlorophyll fluorescence parameters.

## 4 Discussion

### 4.1 Effects of different exogenous substances on yield and agronomic traits of dry-cultivated rice

Spraying exogenous substances is an important strategy for alleviating drought stress in dry-cultivated rice and plays a significant role in the formation of dry-cultivated rice yield. In this study, the combined application of salicylic acid (SA) + silicon (Si) increased yield by 79% compared with the control (CK) treatment. The yield increase was primarily attributed to increases in the number of grains per panicle and the number of effective panicles. Previous studies have found that the positive effects of exogenous application of nano-silicon or proline significantly increased spike length, the number of spikes per plant, the number of grains per spike, grain filling, 1000-grain weight, spike weight, and grain yield (Abd-El-Aty et al., 2024). Maghsoudi et al. (2019) reported that foliar application of SA, Si, and Si + SA significantly increased grain yield under water-limited conditions. The effect of Si + SA was more pronounced than that of Si or SA alone, which is consistent with our results. As a plant growth regulator and a mineral nutrient, respectively, SA and silicon are considered capable of regulating various physiological and biochemical processes in plants, including photosynthesis, stomatal regulation, and ion absorption. Therefore, Si and SA may play important roles in promoting plant growth and increasing yield (Barros et al., 2019). Si can improve plant nutrient uptake and maintain nutrient balance, even under drought stress, which is essential for plant growth and physiological processes (Kaur et al., 2022). SA can also enhance nutrient uptake and transport within plants (Kaur et al., 2022).

Liu et al. (2025) revealed that drought stress affected the growth and development of rice, with plant height, fresh matter weight, and dry matter weight decreasing as drought stress intensified. Spraying SA increased plant height, fresh matter quality, and dry matter quality, with significant increases observed in both fresh matter quality and dry matter quality. Studies have indicated that silicon application under drought stress conditions can regulate the levels of polyamines (PA) and 1-aminocyclopropane-1-carboxylic acid (ACC), thereby promoting root growth and increasing the root-to-stem ratio and root water uptake (Yin et al., 2014). Silicon-mediated changes in root development can also increase the silicification and suppository of root endothelium, thereby enhancing water retention capacity to cope with drought stress (Yin et al., 2014). Si can enhance the water absorption capacity of roots, enabling plants to maintain adequate water status under drought conditions. SA can regulate stomatal closure and reduce water loss through transpiration (Alam et al., 2023). It has been reported that both Si and SA can promote the growth and biomass accumulation of rice plants under cadmium stress alone (Tang et al., 2023). Strong roots are beneficial for crops, as they facilitate the absorption of water and nutrients from the soil and promote plant growth (Gao et al., 2023b). When rice is subjected to drought stress, the roots first perceive the water deficit in the surrounding environment (Yue et al., 2023). This study showed that exogenous SA + Si significantly increased the root length, plant height, shoot dry weight, and root dry weight of the two dry-cultivated rice. Moreover, the combined application of SA + Si was more effective than the single application (**Table 1**; **Figure 2**). Studies have shown that combined application of SA + Si promotes root development and enhances drought tolerance in rice plants, thereby increasing yield (Nadarajah and Kumar, 2019). This may be attributed to the deposition of Si in the cell walls, which increases their thickness and rigidity, thereby providing structural support to plants, particularly under drought stress (Yin et al., 2014). In this study, the yields of the two dry-cultivated rice varieties, SJ18 and HK88, increased by 80.7 and 77.3%, respectively, under SA + Si treatment compared with CK (**Figure 1**). Consistent with previous research findings, the difference in performance between the two varieties may be attributed to their contrasting levels of drought tolerance. SJ18 has strong drought tolerance, while HK88 has weak drought tolerance. Both varieties exhibited positive responses to SA + Si treatment. Although HK88 exhibited numerically greater increases in some physiological traits, the variety × treatment interaction was not significant for most yield and physiological parameters. Therefore, the observed varietal differences should be interpreted as indicative rather than conclusive. The SA + Si treatment may be more effective in compensating for the physiological limitations of drought-sensitive varieties under stress conditions. However, the plant water status (such as leaf relative water content and water potential) or the level of membrane lipid peroxidation was not directly measured in this study. Future studies should therefore incorporate water-related physiological indicators to determine whether SA + Si treatment truly enhances the inherent drought tolerance of different varieties (**Table 2**).

### 4.2 Effects of different exogenous substances on photosynthetic physiology of rice leaves in dry-cultivated rice

Chlorophyll content and fluorescence parameters can reflect the response of total photosynthetic productivity to environmental factors. Therefore, exploring the genetic factors controlling chlorophyll content and fluorescence parameters in rice flag leaves is of great significance for improving the photosynthetic productivity and yield of rice (Gokulraj et al., 2018). Both Si and SA exhibit antioxidant activity, enabling them to scavenge reactive oxygen species and reduce drought-induced oxidative stress. This reduction in oxidative stress can prevent cellular structural damage and maintain plant viability (Wang et al., 2021). According to previous reports, silicon (Si) application increased the size of chloroplasts and the number of grana in crop leaves, thereby increasing their photosynthetic capacity and photosystem II (PSIl) efficiency (Xie et al., 2014). The higher photosynthetic rate may be associated with improvements in water absorption efficiency, stomatal conductance, water use efficiency, and root hydraulic conductance after Si application to rice under drought conditions (Gokulraj et al., 2018). Our study found that the maximum photochemical (quantum) efficiency of photosystem II (PSII) (Fv/Fm) and the photochemical quantum yield (ΦPSII) of dry-cultivated rice increased significantly after Si application. Moreover, Fv/Fm increased more after the Si + SA combined application, reaching a value 25.1% higher than that of CK, which was consistent with previous research findings. It has been reported that SA application can increase cytokinin levels, improve nitrogen absorption and utilization, and enhance the photosynthetic capacity of rice (Talla et al., 2016). Cytokinin is an important hormone involved in cell division and plays a vital role in plant growth and development by promoting growth and enhancing stress tolerance (Comas et al., 2013). In addition, the external application of SA helps alleviate oxidative stress in plants under drought stress by regulating enzymatic and non-enzymatic antioxidant pathways and the glycosidase system (Khan et al., 2015). It also improves root growth parameters, plant height, and leaf area observed under drought conditions. Supplementation with SA in drought-stressed crops has been shown to significantly increase stomatal conductance, thereby enhancing the net CO2 assimilation rate and overall plant growth, including plant dry weight (Khan et al., 2015).

Drought causes a variety of morphological, physiological, and metabolic changes in plants (Urmi et al., 2023) and stimulates the production of ROS in plants. In order to counteract the effects of reactive oxygen species, rice plants have developed antioxidant enzymes, such as superoxide dismutase (SOD), peroxidase (POD), catalase (CAT), glutathione reductase (GR), and ascorbic acid peroxidase (APX). These enzymes scavenge excess reactive oxygen species under various stress conditions, such as salinity, drought, and extreme temperatures (Sun et al., 2016). The combined application of SA and Si to the aboveground parts of *Scrophularia striata* L. was found to enhance the response to drought stress by reducing the content of MDA and H_2_O_2_ (Shohani et al., 2023). In this study, the combined application of SA and Si significantly improved the antioxidant enzymes in the two dry-cultivated rice varieties (**Figure 6**). Increased antioxidant enzyme activity can effectively enhance the antioxidant capacity of dry-cultivated rice and, consequently, improve its ability to regulate drought. This may be attributed to the role of Si supplementation in enhancing antioxidant enzyme activity and protecting plant tissues from membrane oxidative damage under drought stress, thereby contributing to improved drought tolerance of plants (Biju et al., 2017).

Rice has various mechanisms to cope with drought, such as the activation of enzymatic and non-enzymatic antioxidant systems and the accumulation of compatible osmolytes (Mushtaq et al., 2024). Drought affects osmotic adjustment substances such as soluble sugar, which help maintain cell structural stability and enhance drought tolerance by acting as an osmotic protective agent (Khaleghi et al., 2019). In addition, crop drought tolerance is associated with higher soluble protein content, and increasing the accumulation of both can enhance the adaptive drought tolerance of rice (Ozturk et al., 2020). Our previous studies have shown that, under drought conditions, Si helps maintain normal physiological and biochemical metabolism in rice by regulating soluble sugar levels, increasing cytoplasmic concentration, reducing its osmotic potential, and promoting water absorption and the maintenance of cell turgor. The growth of dry-cultivated rice conditions enhances the adaptability of dry-cultivated rice conditions (Jiang et al., 2023). Ali et al. (2021) found that the individual application of SA or Si increased the accumulation of soluble sugars and soluble proteins in rice seedlings. Yang et al. (2021) found that the synergistic effect of SA and Si enhanced the accumulation of soluble sugars and soluble proteins, thereby reducing chromium toxicity in rice. In this study, the combined application of SA + Si significantly increased soluble sugar content in SJ18 and HK88 (**Figure 5**). After 10 days of spraying, soluble sugar content of SJ18 and HK88 increased by 10.7 and 36.1%, respectively, compared with CK, while soluble protein content increased by 17.4 and 22.6%, respectively. The accumulation of osmotic adjustment substances in HK88 was significantly greater than that in SJ18. Under the specific experimental conditions used in this study, exogenous application of SA + Si significantly increased the levels of soluble sugars and soluble proteins in dry-cultivated rice, thereby enhancing its drought tolerance. This effect was particularly pronounced in the dry-cultivated rice varieties with lower drought tolerance.

### 4.3 SA + Si combined application was associated with increased photosynthetic capacity, antioxidant activity, and yield in dry-cultivated rice

The application of SA + Si significantly improved the growth, photosynthetic pigments, and chlorophyll fluorescence parameters of maize seedlings. It also maintained membrane stability by reducing reactive oxygen species and lipid damage and significantly alleviated the cadmium-induced toxic effects (Singh et al., 2019). We found that the combination of SA and Si significantly increased the maximum photochemical efficiency of PSII (**Table 2**). When plant cells were subjected to stress, membrane permeability and thylakoid function in chloroplasts were damaged, resulting in a gradual decline in photosystem activity and chlorophyll fluorescence. Under stress conditions, the repair of plant photosystem II is affected, photoinhibition is enhanced, and photosynthetic efficiency is reduced (Chen et al., 2024). Our study also showed that the Fv/Fm values of T1 (Si), T2 (SA), and T3 (SA + Si) treatments of the two varieties increased by an average of 13.2%, 20.2%, and 25.1%, respectively, compared with CK treatment (**Table 2**). Among the treatments, the combined application of SA and Si resulted in the greatest increase in Fv/Fm values. Comprehensive physiological, anatomical, and phytochemical results showed that drought stress increased oxidative damage, whereas the application of SA and Si under drought conditions significantly alleviated these adverse effects by enhancing antioxidant activity, increasing photosynthetic pigment content and relative water status, and maintaining anatomical integrity. In our study, we also found that there was a significant positive correlation between yield and yield components (except seed-setting rate), plant height, primary light energy conversion efficiency, actual photochemical quantum yield, and endogenous Si content in every part of the plant.

Aboveground and underground dry matter weight was mainly affected by plant height, root length, primary light energy conversion efficiency, actual photochemical quantum yield, soluble protein content, and endogenous Si content in different parts of the plant (**Figure 8**). Under drought conditions, SA and Si did not evenly increase the accumulation of metabolites. Rather, they regulated the composition and relative abundance of specific metabolite classes in a treatment- and ecotype-dependent manner (Shohani and Fazeli, 2025). In the study of cotton, it was found that the application of SA and Si had significant effects on root length, stem weight, and the root-to-stem ratio, with the combined application of SA and Si producing the greatest effects. According to other reports, the application of SA and Si reduced oxidative parameters, such as H_2_O_2_ and malondialdehyde (MDA), while increasing relative water content and the root-to-shoot ratio by enhancing the activities of enzymatic and non-enzymatic antioxidants (Shohani et al., 2023). Similarly, we found that the antioxidant enzyme activity index was positively correlated with chlorophyll fluorescence parameters (**Figure 8**).

Based on the correlation analysis derived from our structural equation model (SEM), elevated levels of endogenous SA and Si were significantly positively correlated with increased antioxidant enzyme activities (r = 0.835). Improvements in chlorophyll fluorescence parameters were also closely associated with yield enhancement (r = 0.754), while soluble sugar and protein contents were positively correlated (r = 0.244). This may be because the combined application of Si and SA increased endogenous Si and SA contents, which were further associated with higher antioxidant enzyme activity and osmotic adjustment substance content. Antioxidant enzyme activity was positively associated with chlorophyll fluorescence parameters, which, in turn, were positively associated with yield, suggesting that antioxidant enzymes influence yield indirectly through photosynthetic improvement rather than through a direct pathway (**Figure 9**). The observed physiological responses may also involve changes in the regulation of stress-responsive genes. Future transcriptomic or molecular studies could therefore determine whether such transcriptional changes underlie the observed responses.

In addition, both the duration and severity of stress significantly affect the rate and pattern of changes in gene expression (Morshedloo et al., 2016). Reduced gene expression levels may affect the synthesis of primary and secondary metabolites (Yang et al., 2020). The combination of Si and SA may exert a synergistic effect, with each component complementing the other and providing greater benefits than either treatment alone (Barros et al., 2019). Both Si and SA can regulate the expression of stress-responsive genes, thereby activating multiple pathways involved in drought tolerance (Kaur et al., 2022). Therefore, the appropriate combined application of Si and SA can protect the photosynthetic apparatus from drought-induced damage by enhancing antioxidant defense and osmotic regulation, thereby maintaining higher photosynthetic capacity and alleviating drought stress to increase yield. Future experiments involving a wider range of SA concentrations are needed to determine the optimal SA concentration for dry-cultivated rice under drought stress.

It should be noted that drought tolerance in this study was inferred from osmotic adjustment substances, antioxidant enzyme activities, chlorophyll fluorescence, and yield-related parameters. Direct indicators of plant water status (e.g., leaf relative water content, water potential) and membrane oxidative damage (e.g., MDA, H_2_O_2_) were not measured. Therefore, the conclusions regarding drought tolerance should be interpreted as associations rather than definitive causal effects. Although our SEM analysis revealed strong synergistic correlations among these physiological parameters, the proposed regulatory cascade—from osmotic adjustment and endogenous SA/Si accumulation to antioxidant defense and photosynthetic improvement—remains a mechanistic hypothesis based on correlative evidence. Definitive causal relationships and the underlying molecular networks require further validation using genetic approaches, such as gain- or loss-of-function mutants, and targeted transcriptomic/proteomic analyses.

## 5 Conclusion

The combined application of salicylic acid (SA, 1.0 mM) + silicon (Si, 45 kg ha^-1^) significantly increased the dry matter accumulation and yield of two dry-cultivated rice varieties. These yield improvements were accompanied by higher contents of osmotic adjustment substances, endogenous SA and Si, upregulated antioxidant enzyme activities, and enhanced photosynthetic capacity of the PSII system. Structural equation modeling further revealed that these physiological parameters were strongly and positively correlated, suggesting that they may act synergistically to alleviate the inhibition of photosynthesis caused by drought stress and promote yield formation. Varietal differences were observed in the responses to the combined application of SA and Si, with the drought-sensitive variety HK88 showing more pronounced improvements in several physiological traits than the drought-resistant variety SJ18. Collectively, these findings suggest that the rational combined application of SA and Si is a promising strategy. However, this conclusion remains a hypothesis based on correlative evidence and requires further validation through molecular and genetic approaches.

## Data Availability

The original contributions presented in the study are included in the article/supplementary material. Further inquiries can be directed to the corresponding authors.
